# Age-specific association between remnant cholesterol and insulin resistance in women with polyendocrine metabolic ovarian syndrome

**DOI:** 10.3389/fendo.2026.1932335

**Published:** 2026-09-30

**Authors:** Chiyuan Yu, Zhonghua Sun, Qinyao Wu, Zhiyi Zhou, Wenxin Chen, Boqun Xu

**Affiliations:** 1 Women’s Hospital of Nanjing Medical University, Nanjing Women and Children’s Healthcare Hospital, Nanjing, Jiangsu, China; 2 Medical School, Nanjing University, Nanjing, China

**Keywords:** age-specific association, insulin resistance, metabolic risk, polyendocrine metabolic ovarian syndrome, remnant cholesterol

## Abstract

**Background:**

Insulin resistance (IR) is a major metabolic feature in women with polyendocrine metabolic ovarian syndrome (PMOS). Remnant cholesterol (RC), which reflects the cholesterol burden of triglyceride-rich remnant lipoproteins (TRLs), may capture lipid disturbances related to insulin-resistant dyslipidemia. Whether RC is linked to IR in women with PMOS remains unclear.

**Methods:**

This study enrolled 617 women with PMOS. Participants were grouped according to natural-log-transformed remnant cholesterol (lnRC) quartiles. Adjusted odds ratios (ORs) for IR were estimated using multivariable logistic regression models, and restricted cubic splines (RCS) were fitted to characterize the potential dose-response patterns. Multiplicative and additive interaction analyses were performed to evaluate the interaction between age and lnRC. Discrimination of IR by lnRC was assessed using receiver operating characteristic (ROC) curve, and age-specific estimates were compared using DeLong’s test. Integrated discrimination improvement (IDI) and net reclassification improvement (NRI) were calculated to assess whether lnRC added information beyond the base model.

**Results:**

Higher lnRC levels were significantly associated with increased odds of IR (Q4 vs. Q1: odds ratio [OR] = 3.11, 95% confidence interval [CI]: 1.57–6.14, *P* = 0.001). This relationship was stronger in women aged < 30 years than in those aged ≥ 30 years (OR: 2.01 vs. 1.13, *P* for interaction = 0.046). A multiplicative interaction between age and lnRC was also observed for IR (OR = 0.438, 95% CI: 0.197-0.977, *P* = 0.044). Exploratory age-stratified ROC analysis showed that the area under the curve (AUC) of lnRC for IR discrimination was higher among women aged <30 years than among those aged ≥30 years (0.654 vs. 0.523, DeLong *P* = 0.024). Adding lnRC to the base model improved risk reclassification and discrimination (NRI = 0.381, 95% CI: 0.165–0.597, *P* = 0.001; IDI = 0.025, 95% CI: 0.007–0.0439, *P* = 0.005).

**Conclusions:**

This study indicates that the lnRC–IR association was stronger in women aged <30 years, and a multiplicative interaction between lnRC and age group was observed. These findings suggest that RC may provide complementary information for metabolic assessment in younger women with PMOS and support its further evaluation as a readily calculable lipid marker.

## 1 Introduction

Polyendocrine metabolic ovarian syndrome (PMOS), formerly known as polycystic ovary syndrome (PCOS), has recently been put forward as a revised name for this complex disorder. The change in terminology is intended to lessen the possible misunderstanding created by the word “polycystic” (1), while giving greater emphasis to the broader clinical picture, including reproductive endocrine dysfunction, insulin-related metabolic disturbances, ovarian dysfunction, and long-term health risks. Among women of reproductive age, PMOS represents one of the most prevalent endocrine and metabolic disorders, affecting an estimated 12.5% of this population worldwide (1).

Compared with women in the general population, those with PMOS have a higher susceptibility to metabolic syndrome, and clinical manifestations such as obesity, dyslipidemia, impaired glucose tolerance, hyperglycemia, and hypertension are frequently observed (2–4). Among these abnormalities, insulin resistance (IR) is a key metabolic feature of PMOS (5–7). It is closely linked to hyperinsulinemia, ovulatory dysfunction, and hyperandrogenism, and it may also drive later metabolic complications (5, 8–10). Lipid metabolic disturbance is another important part of the insulin-resistant phenotype in PMOS. Around 70% of patients are affected, typically showing higher triglycerides (TG), lower high-density lipoprotein cholesterol (HDL-C), and other atherogenic lipid abnormalities (11–13). PMOS is characterized by systemic metabolic and endocrine dysfunction involving multiple organs and tissues. Recent studies have highlighted the central roles of the liver and adipose tissue, both of which are major sites of lipid metabolism, in the pathophysiology of PMOS. Disturbances in lipid metabolism and insulin resistance may interact bidirectionally, thereby contributing to the broader metabolic and endocrine abnormalities associated with PMOS (14). Previous studies in PMOS have linked the triglyceride–glucose (TyG) index, atherogenic index of plasma (AIP), and ratios of LDL-C, TC, or TG to HDL-C to IR and unfavorable metabolic phenotypes (12, 15, 16). However, routine lipid concentrations and simple lipid ratios do not always capture the lipoprotein-related metabolic burden in this population.

In this context, clinical research has increasingly focused on remnant cholesterol (RC) as a potentially informative lipid measure. RC reflects the cholesterol content of remnant triglyceride-rich lipoproteins (TRLs). Remnant TRLs comprise chylomicron remnants, very-low-density lipoprotein (VLDL) remnants, and intermediate-density lipoprotein (IDL) (17, 18). Evidence from multiple multicenter clinical studies indicates that RC can independently predict several diseases, including atherosclerotic cardiovascular disease, venous thromboembolism, ischemic stroke, and type 2 diabetes mellitus (19–23). Moreover, individuals with prolonged exposure to increased RC levels tend to have a greater risk of developing IR (24–27). In PMOS, higher RC levels have also been observed in metabolically high-risk subgroups, including women with coexisting metabolic dysfunction-associated steatotic liver disease (MASLD) or obesity (28, 29). However, these studies have focused on cardiometabolic risks and have included relatively small samples. Thus, the independent relationship between RC and IR in a broader PMOS population still needs further clarification.

Therefore, we conducted the present study to investigate the relationship between RC and IR among women with PMOS. We also explored age-related heterogeneity, aiming to assess whether RC may be particularly informative in younger women with PMOS. These findings warrant further investigation of RC as an easily calculated marker that may complement conventional metabolic assessment.

## 2 Materials and methods

### 2.1 Study design and patients

This observational study was carried out at the Department of Gynaecology, Women’s Hospital of Nanjing Medical University, from 1 September 2024 to 3 April 2026. The Medical Ethics Committee of Nanjing Women and Children’s Healthcare Hospital granted approval for the study protocol (Approval No. PJ-2026KY054-002).

PMOS was identified using the Rotterdam criteria. A diagnosis was established when any two of three components were present: oligo-ovulation or anovulation, hyperandrogenism documented clinically or biochemically, sonographic evidence of polycystic ovarian morphology (30). Disorders that might account for hyperandrogenism or ovulatory abnormalities were ruled out before confirmation.

Female participants between 18 and 40 years of age were considered eligible only when all fasting lipid measurements were available. Values for TG, HDL-C, total cholesterol (TC), and low-density lipoprotein cholesterol (LDL-C) were then used to calculate RC.

The exclusion criteria included: (1) other gynecological disorders associated with ovulatory dysfunction, such as endometriosis, primary ovarian insufficiency, uterine fibroids, and related conditions; (2) endocrine disorders affecting hormonal or lipid metabolism, including thyroid disease, Cushing’s syndrome, or adrenal disorders; (3) severe hepatic or renal dysfunction, cardiovascular or cerebrovascular disease, hematological disorders, or malignant tumors; and (4) pregnancy or lactation.

### 2.2 Covariate

Baseline characteristics and medical history were obtained from medical records. These data included age, reproductive endocrine profiles (LH, FSH, E2, T, P4, AMH, and SHBG), markers of glucose metabolism (FBG, INS0, and HbA1c), and serum lipid parameters (TG, TC, HDL-C, and LDL-C). We also collected anthropometric measurements, including body weight, body mass index (BMI), waist circumference (WC), hip circumference (HC), and waist-to-hip ratio (WHR), along with routine biochemical and hematological parameters.

The derived indices were calculated as follows: BMI = body weight (kg)/height (m)²; WHR =WC (cm)/HC (cm).

### 2.3 Exposures

RC was treated as the principal exposure variable and reflected the cholesterol content carried by TRLs. Values were derived from the conventional lipid panel using the equation RC = TC - LDL-C - HDL-C. Because RC showed a skewed distribution, its values were transformed on the natural logarithmic scale before analysis, and the resulting variable was termed lnRC. Serum TC, LDL-C, and HDL-C were quantified by routine enzymatic assays.

### 2.4 Outcome

IR status was determined from Homeostasis Model Assessment of Insulin Resistance (HOMA-IR), calculated as fasting insulin (μIU/mL) × fasting glucose (mmol/L)/22.5 (31, 32). IR was defined as a HOMA-IR value of ≥ 2.5 (33).

After an overnight fast of at least 8 h, venous samples were collected and analyzed in the hospital’s central laboratory using standardized assay procedures.

### 2.5 Statistical analysis

The study population was stratified into four categories using lnRC quartile cutoffs. Continuous data are described using the mean and standard deviation when their distributions were approximately normal; otherwise, the median and interquartile range are reported. Differences among lnRC quartile groups were examined using one-way ANOVA or the Kruskal-Wallis test. Logistic regression analyses were constructed to quantify the relation of lnRC to IR, with lnRC entered as either a continuous measure or a quartile-based categorical variable. A crude model was first fitted, followed by progressively adjusted multivariable models (age, BMI, and testosterone). To characterize the shape of the lnRC–IR association, we applied restricted cubic spline (RCS) model and assessed evidence of nonlinearity. Subgroup analyses were performed after stratification by age and BMI, testosterone, SHBG, AMH, and LH/FSH ratio. Age-stratified analyses were performed using a 30-year cutoff (<30 vs. ≥30 years), based on age categorizations used in previous PMOS studies (34–37). To assess the robustness, the analyses were repeated using 35 years as an alternative cutoff in sensitivity analyses. Multiplicative and additive interaction analyses were conducted to determine whether age modified the lnRC–IR relationship. To examine additive interaction, lnRC was dichotomized at its median. Relative excess risk due to interaction (RERI), attributable proportion (AP), and synergy index (SI) were derived from the estimated odds ratios, with confidence intervals obtained using the delta method. To assess the discriminatory performance of lnRC for IR, receiver operating characteristic (ROC) curves were generated for the overall population and age group. Given the potential influence of BMI on the interaction between age and lnRC, we further examined the three-way interaction (lnRC × age × BMI) in relation to IR. Differences in the area under the curve (AUC) were evaluated with DeLong’s method. Integrated discrimination improvement (IDI) and net reclassification improvement (NRI) were used to assess the incremental contribution of lnRC over the base model. Sensitivity analyses were performed by repeating the analyses after multiple imputation of missing testosterone measurements and by applying an alternative HOMA-IR threshold of 2.69 (38). Statistical computations were completed in R, version 4.4.1. *P*< 0.05 was considered statistically significant.

## 3 Results

### 3.1 Clinical and biochemical profiles by lnRC category

**Figure 1** illustrated the process of participant selection. After eligibility screening, 617 women with PMOS remained in the final analysis, and 218 (35.3%) of them were identified as having IR. Clinical and biochemical profiles across the four lnRC categories are summarized in **Table 1**. The cutoff values were specified as follows: Quartile 1 (Q1), ≤ 2.23; Quartile 2 (Q2), 2.23-2.81; Quartile 3 (Q3), 2.81-3.18; and Quartile 4 (Q4), ≥ 3.18.

**Figure 1:**
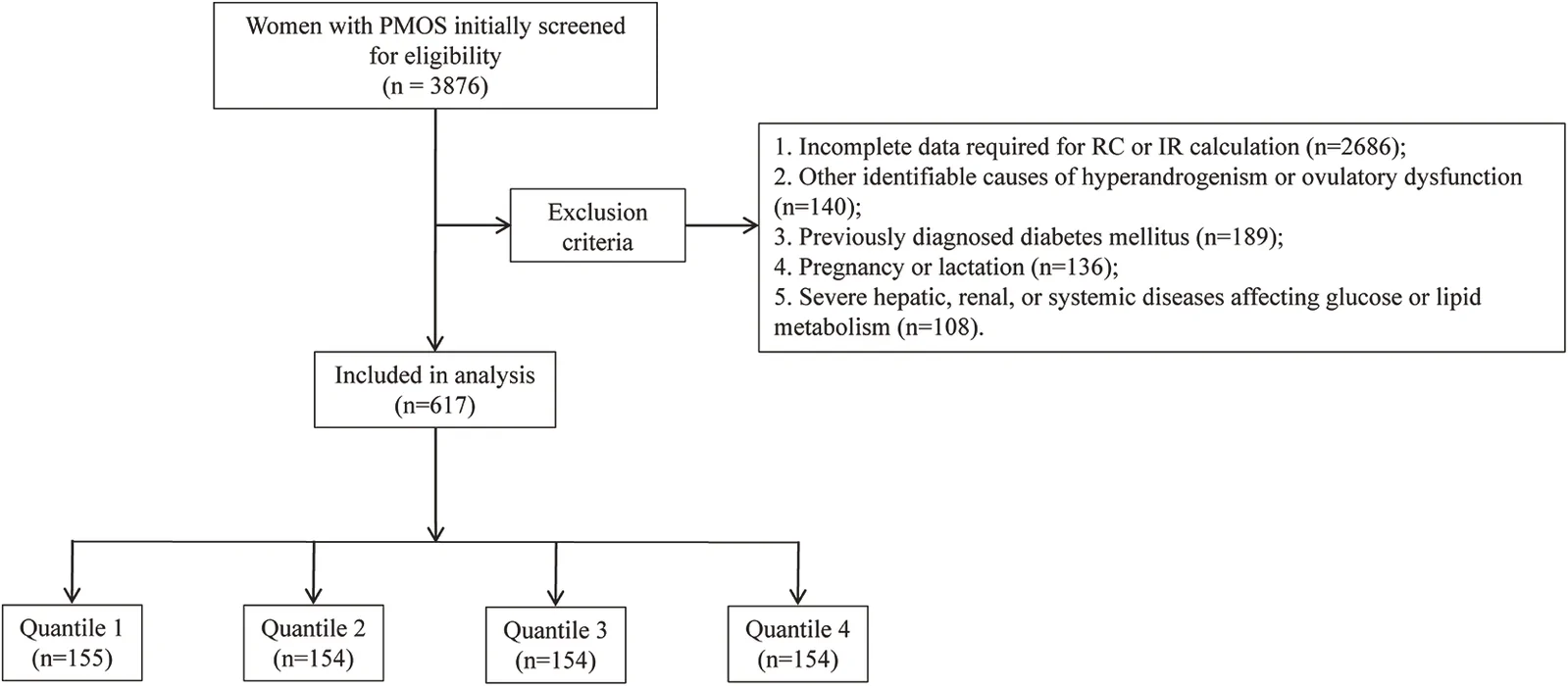
Flowchart of study participants.

**Table 1:** Baseline characteristics of the participants according to LnRC quartiles in PMOS.

Variables	Total (n=617)	Q1 (n=155)	Q2 (n=154)	Q3 (n=154)	Q4 (n=154)	*P-*value
Age (year)	25.0 (22.0, 29.0)	24.0 (22.0, 28.0)	25.0 (22.0, 28.0)	26.0 (22.0, 29.0)	26.0 (22.0, 30.0)	0.123
BMI (kg/m^2^)	23.7 (21.1, 26.9)	22.8 (20.1, 25.5)	22.7 (20.4, 26.7)	24.0 (21.2, 27.2)	25.0 (22.7, 27.6)	< 0.001
WC (cm)	80.8 (72.0, 89.2)	72.2 (69.7, 81.2)	76.4 (70.8, 88.2)	82.0 (73.1, 89.2)	81.8 (79.1, 92.6)	0.001
HC (cm)	96.0 (91.0, 101)	91.0 (88.0, 97.5)	95.0 (88.8, 100)	98.0 (92.0, 105)	96.0 (92.5, 104)	0.012
WHR	0.86 ± 0.07	0.86 ± 0.08	0.85 ± 0.06	0.86 ± 0.07	0.87 ± 0.06	0.326
AMH (ng/ml)	9.93 (7.22, 13.6)	9.85 (7.26, 13.2)	9.23 (6.22, 13.8)	9.34 (6.64, 12.4)	11.0 (8.23, 15.2)	0.083
Basal FSH (mIU/mL)	6.00 ± 1.97	5.74 ± 2.00	6.00 ± 1.98	6.09 ± 2.03	6.18 ± 1.86	0.399
Basal LH (mIU/mL)	13.5 (8.13, 18.7)	13.5 (7.76, 18.5)	13.8 (7.47, 19.6)	13.3 (8.43, 17.8)	13.1 (9.10, 17.9)	0.903
Basal PRL (ng/mL)	12.4 (9.53, 18.8)	12.3 (9.45, 18.3)	12.6 (9.20, 19.0)	12.9 (9.83, 19.6)	12.4 (9.33, 16.2)	0.709
Basal E2 (pg/mL)	45.4 (34.4, 63.7)	49.4 (38.0, 69.1)	51.9 (37.0, 78.8)	42.4 (31.5, 55.9)	42.2 (31.4, 53.7)	0.001
Basal T (pg/mL)	0.49 (0.35, 0.70)	0.48 (0.36, 0.68)	0.48 (0.34, 0.70)	0.49 (0.35, 0.66)	0.51 (0.36, 0.74)	0.831
Basal P4 (ng/mL)	0.61 (0.38, 1.13)	0.65 (0.43, 1.45)	0.68 (0.42, 1.27)	0.57 (0.34, 0.92)	0.57 (0.33, 1.03)	0.038
SHBG (nmol/L)	26.7 (16.8, 44.0)	27.3 (18.4, 46.9)	32.2 (20.0, 50.6)	26.1 (16.4, 42.7)	22.9 (14.9, 31.4)	0.004
FAI	1.50 (0.92, 2.41)	1.35 (0.90, 2.30)	1.32 (0.72, 2.18)	1.32 (0.83, 2.24)	1.90 (1.21, 2.84)	0.015
TSH (mIU/L)	2.17 (1.48, 2.95)	2.11 (1.44, 2.91)	2.29 (1.51, 3.47)	2.20 (1.55, 2.88)	2.29 (1.47, 3.04)	0.762
FT4 (pmol/L)	17.0 ± 2.29	16.9 ± 2.31	16.4 ± 2.46	17.4 ± 3.00	17.3 ± 1.08	0.856
TC (mg/dL)	182.9 (164.0, 201.1)	169.3 (152.8, 188.3)	172.1 (156.7, 192.2)	182.9 (167.8, 196.8)	208.4 (186.8, 233.2)	< 0.001
TG (mg/dL)	9.12 (6.37, 13.6)	6.81 (5.31, 10.1)	7.88 (6.11, 11.9)	9.65 (7.01, 13.5)	13.9 (9.23, 20.3)	< 0.001
HDL-C (mg/dL)	57.2 (47.6, 67.7)	61.9 (51.4, 71.5)	59.0 (49.2, 68.8)	56.5 (46.1, 65.0)	52.0 (43.8, 63.7)	< 0.001
LDL-C (mg/dL)	106 (89.7, 125)	101 (82.2, 119)	97.3 (82.5, 116)	108 (93.6, 121)	120 (100, 140)	< 0.001
FBG (mmol/L)	5.06 (4.81, 5.35)	5.04 (4.78, 5.28)	4.98 (4.81, 5.31)	5.07 (4.84, 5.36)	5.12 (4.84, 5.45)	0.059
INS0 (uIU/mL)	8.53 (5.71, 13.2)	6.92 (5.11, 10.2)	8.51 (5.41, 12.2)	9.01 (5.82, 13.8)	10.6 (6.96, 16.8)	< 0.001
HOMA-IR	1.91 (1.28, 3.02)	1.57 (1.10, 2.32)	1.90 (1.19, 2.81)	2.04 (1.35, 3.09)	2.40 (1.60, 3.88)	< 0.001
HbA1c%	5.30 (5.10, 5.50)	5.30 (5.10, 5.50)	5.30 (5.00, 5.43)	5.30 (5.20, 5.50)	5.40 (5.00, 5.50)	0.836
AST (U/L)	19.2 (16.8, 23.3)	18.2 (16.1, 21.0)	18.8 (16.8, 21.0)	20.0 (17.2, 23.6)	20.4 (18.0, 26.9)	< 0.001
ALT (U/L)	17.5 (12.4, 29.2)	15.8 (11.0, 23.9)	16.6 (12.2, 26.0)	18.4 (12.9, 31.0)	22.3 (13.6, 38.0)	< 0.001
GGT (U/L)	17.9 (13.8, 27.2)	17.2 (13.7, 25.9)	14.8 (12.7, 21.8)	18.8 (14.3, 31.1)	20.1 (15.8, 28.2)	0.003
UA (umol/L)	300 (257, 350)	305 (272, 350)	282 (233, 329)	294 (259, 347)	319 (265, 383)	0.009
Cr (umol/L)	50.0 ± 8.32	52.7 ± 8.06	48.9 ± 8.04	49.4 ± 8.76	48.6 ± 7.76	< 0.001
Urea (mmol/L)	4.50 (3.82, 5.30)	4.60 (3.87, 5.28)	4.24 (3.76, 5.08)	4.40 (3.78, 5.04)	4.88 (3.99, 5.75)	0.002
TP (g/L)	46.5 ± 2.53	47.0 ± 2.16	46.5 ± 2.46	46.0 ± 2.53	46.5 ± 2.90	0.073
ALB (g/L)	75.2 (72.3, 77.6)	75.0 (72.2, 76.8)	74.3 (72.2, 77.0)	75.6 (73.0, 77.8)	76.2 (72.6, 78.5)	0.316
Hb (g/L)	135 (128, 140)	136 (129, 140)	132 (122, 138)	134 (128, 137)	141 (133, 142)	0.046
PLT (10^9/L)	266 (232, 314)	263 (237, 327)	244 (218, 302)	284 (230, 334)	266 (240, 282)	0.571
WBC (10^9/L)	6.16 (5.52, 7.39)	5.97 (5.32, 7.21)	5.75 (5.43, 7.01)	6.67 (5.60, 7.86)	6.13 (5.68, 7.22)	0.446
LYM (10^9/L)	2.18 ± 0.58	2.23 ± 0.62	1.91 ± 0.48	2.32 ± 0.38	2.20 ± 0.74	0.006

Data are expressed as median, mean ± SD (interquartile range) for continuous variables.

lnRC, natural log-transformed remnant cholesterol; BMI, body mass index; WC, waist circumference; HC, hip circumference; WHR, waist-to-hip ratio; AMH, anti-Müllerian hormone; FSH, follicle-stimulating hormone; LH, luteinizing hormone; PRL, prolactin; E2, estradiol; T, testosterone; P4, progesterone; SHBG, sex hormone-binding globulin; FAI, free androgen index; TSH, thyroid-stimulating hormone; FT4, free thyroxine 4; TC, total cholesterol; TG, triglycerides; HDL-C, high-density lipoprotein cholesterol; LDL-C, low-density lipoprotein cholesterol; FBG, fasting blood glucose; INS0, fasting insulin; HOMA-IR, homeostatic model assessment of insulin resistance; HbA1c, glycated hemoglobin A1c; AST, aspartate aminotransferase; ALT, alanine aminotransferase; GGT, gamma-glutamyl transferase; UA, uric acid; Cr, creatinine; Urea, urea nitrogen; TP, total protein; ALB, albumin; Hb, hemoglobin; PLT, platelets; WBC, white blood cell; LYM, lymphocyte.

Across increasing lnRC quartiles, adiposity-related indices, including BMI, WC, and HC, tended to rise. Fasting insulin and HOMA-IR followed the same upward pattern. Lipid metabolism also became more adverse, TG, TC, and LDL-C rose, whereas HDL-C declined. For endocrine-related parameters, SHBG levels declined and FAI levels increased, while LH, testosterone, and AMH did not show clear increasing trends. Patients in the higher lnRC quartiles also had relatively higher AST, ALT, GGT, uric acid, creatinine, and urea levels.

### 3.2 Association between lnRC and IR in women with PMOS

**Table 2** presented the relationship between lnRC and IR. After adjustment for multiple covariates, the odds of IR increased by 59% for every 1-unit rise in lnRC (OR = 1.59, 95% CI: 1.18–2.12, *P* = 0.002). When lnRC was analyzed by quartiles, higher lnRC quartiles (Q2–Q4) were consistently associated with the presence of IR compared with Q1 across all three models, and the trend across quartiles remained statistically significant. Notably, Women in Q4 had 3.11 times the odds of IR compared with those in Q1 (OR = 3.11, 95% CI: 1.57–6.14, *P* = 0.001).

**Table 2:** Association between lnRC and the risk of IR in PMOS.

Categories	Crude model		Model 1		Model 2		Model 3	
	OR (95% CI)	*P* value	OR (95% CI)	*P* value	OR (95% CI)	*P* value	OR (95% CI)	*P* value
lnRC	1.75 (1.37-2.21)	< 0.001	1.72 (1.36-2.19)	< 0.001	1.67 (1.28-2.16)	< 0.001	1.59 (1.18-2.12)	< 0.001
lnRC (quartiles)								
Q1 (≤ 2.23)	1.00 (reference)		1.00 (reference)		1.00 (reference)		1.00 (reference)	
Q2 (2.23-2.81)	1.96 (1.17-3.27)	0.01	1.95 (1.16-3.25)	0.011	2.36 (1.28-4.35)	0.006	2.05 (1.04-4.05)	0.039
Q3 (2.81-3.18)	2.39 (1.44-3.96)	< 0.001	2.35 (1.41-3.91)	< 0.001	2.63 (1.42-4.89)	0.002	3.04 (1.52-6.09)	0.002
Q4 (≥ 3.18)	3.65 (2.21-6.02)	< 0.001	3.56 (2.15-5.89)	< 0.001	3.19 (1.75-5.81)	< 0.001	3.11 (1.57-6.14)	0.001
*P* for trend	< 0.001		< 0.001		< 0.001		0.003	

OR, Odds Ratio; CI, Confidence Interval.

Crude model: unadjusted.

Model 1: adjusted for age.

Model 2: adjusted for age, BMI.

Model 3: adjusted for age, BMI, Testosterone.

### 3.3 Analysis of the dose-response relationship

A four-knot RCS model was fitted to evaluate the dose-response relationship between lnRC and IR, using the median lnRC value (2.788) as the reference point (**Figure 2**). lnRC showed a significant overall association with IR (*P* for overall = 0.02), whereas no evidence of nonlinearity was detected (*P* for nonlinearity = 0.651). The estimated curve showed a generally upward trend, with higher lnRC levels corresponding to progressively higher odds of IR.

**Figure 2:**
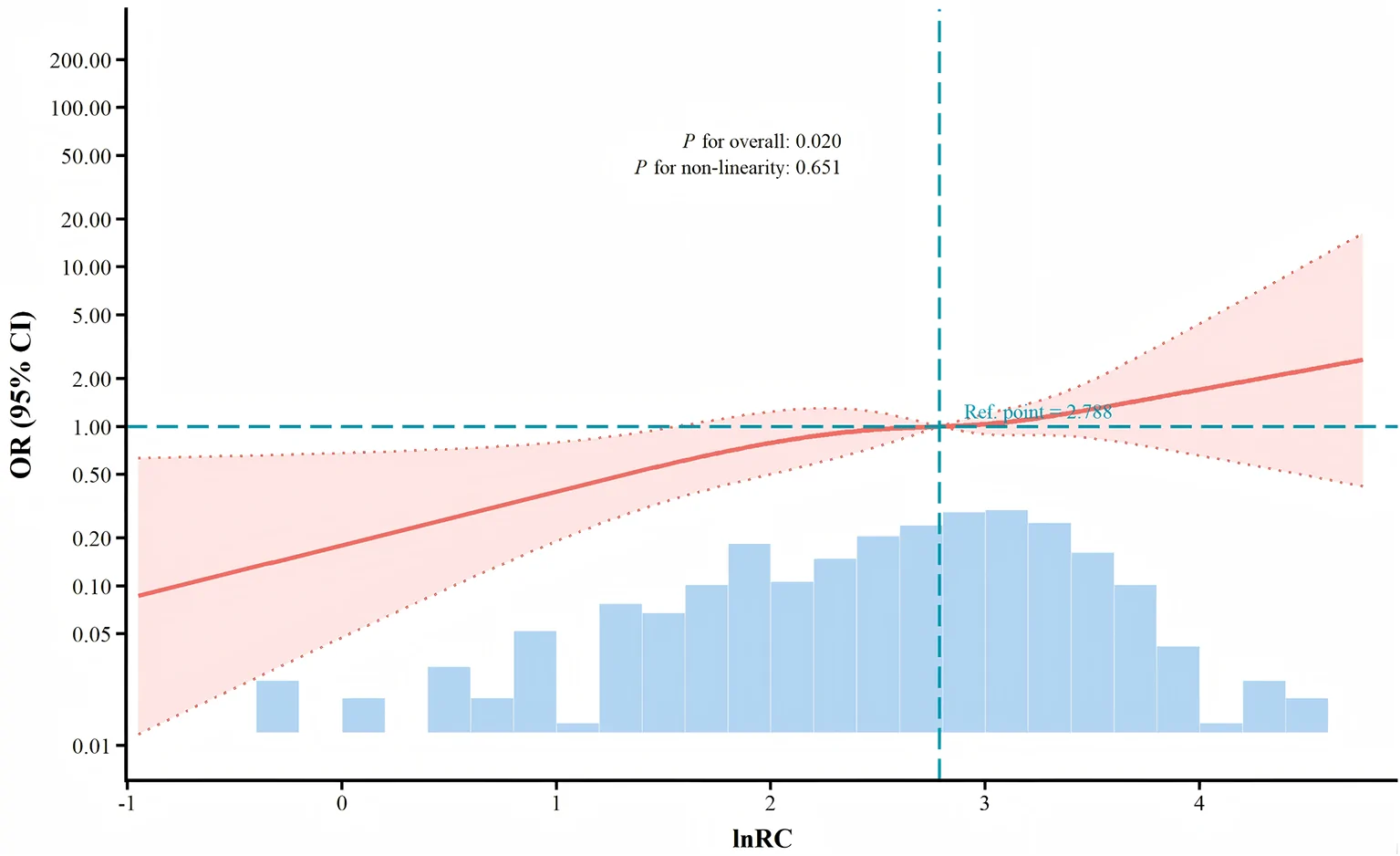
Dose-response association between lnRC and IR in PMOS. Adjusted for age, BMI and testosterone.

### 3.4 Age-specific association and discriminative performance of lnRC for IR

To explore the potential influence of age, BMI, and sex hormone levels, findings from the subgroup analyses are presented in **Figure 3**. The positive relationship between lnRC and IR was generally consistent across most subgroups, including the BMI categories (<24, 24 to <28, and ≥28 kg/m²), with no statistically significant evidence of interaction (*P* for interaction = 0.582). Age-specific models yielded different estimates for lnRC (*P* for interaction = 0.046). For each 1-unit increase in lnRC, the OR for IR was 2.01 (95% CI: 1.51–2.67; *P* < 0.001) among women younger than 30 years, whereas the corresponding OR was 1.13 (95% CI: 0.70–1.82; *P* = 0.61) among women aged 30 years or older. Consistent with the subgroup findings, the lnRC–IR association showed age-related heterogeneity on the multiplicative scale (OR = 0.438, 95% CI: 0.197–0.977, *P* = 0.044), indicating attenuation of the relationship in the older age group (**Table 3**). However, no statistically significant additive interaction was observed.

**Figure 3:**
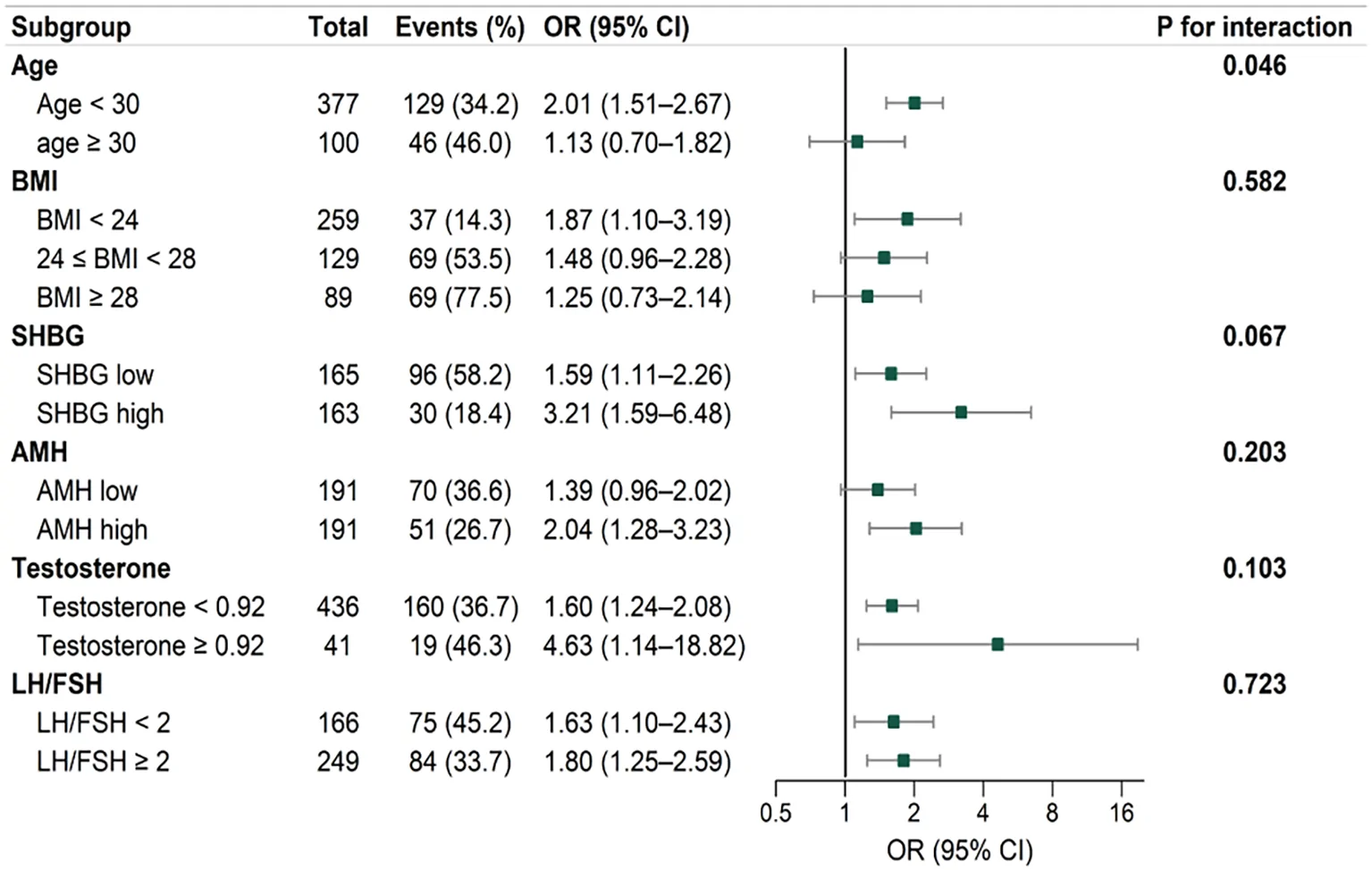
Subgroup analysis of the association between lnRC and IR risk.

**Table 3:** Multiplicative and additive interactions between age and lnRC in relation to IR.

Measures	Estimates (95% CI)	*P*-value
Multiplicative interaction		
Multiplicative scale	0.438 (0.197-0.977)	0.044
Additive interaction		
RERI (95% CI)	-1.212 (-2.960-0.536)	0.174
AP (95% CI)	-0.541 (-1.452-0.370)	0.245
SI (95% CI)	0.506 (0.191-1.337)	0.169

RERI, relative excess risk due to interaction; AP, attributable proportion due to interaction; SI, synergy index.

This pattern was also reflected in exploratory ROC analysis (**Figure 4**), where lnRC showed better discriminatory performance in women aged < 30 years than in the older group (AUC: 0.654 vs. 0.523), with a significant difference according to the DeLong test (*P* = 0.024). The corresponding optimal cutoff values were 2.434 and 2.288, respectively (**Table 4**). In the overall population, lnRC had limited discriminatory ability for identifying IR (AUC = 0.627).

**Figure 4:**
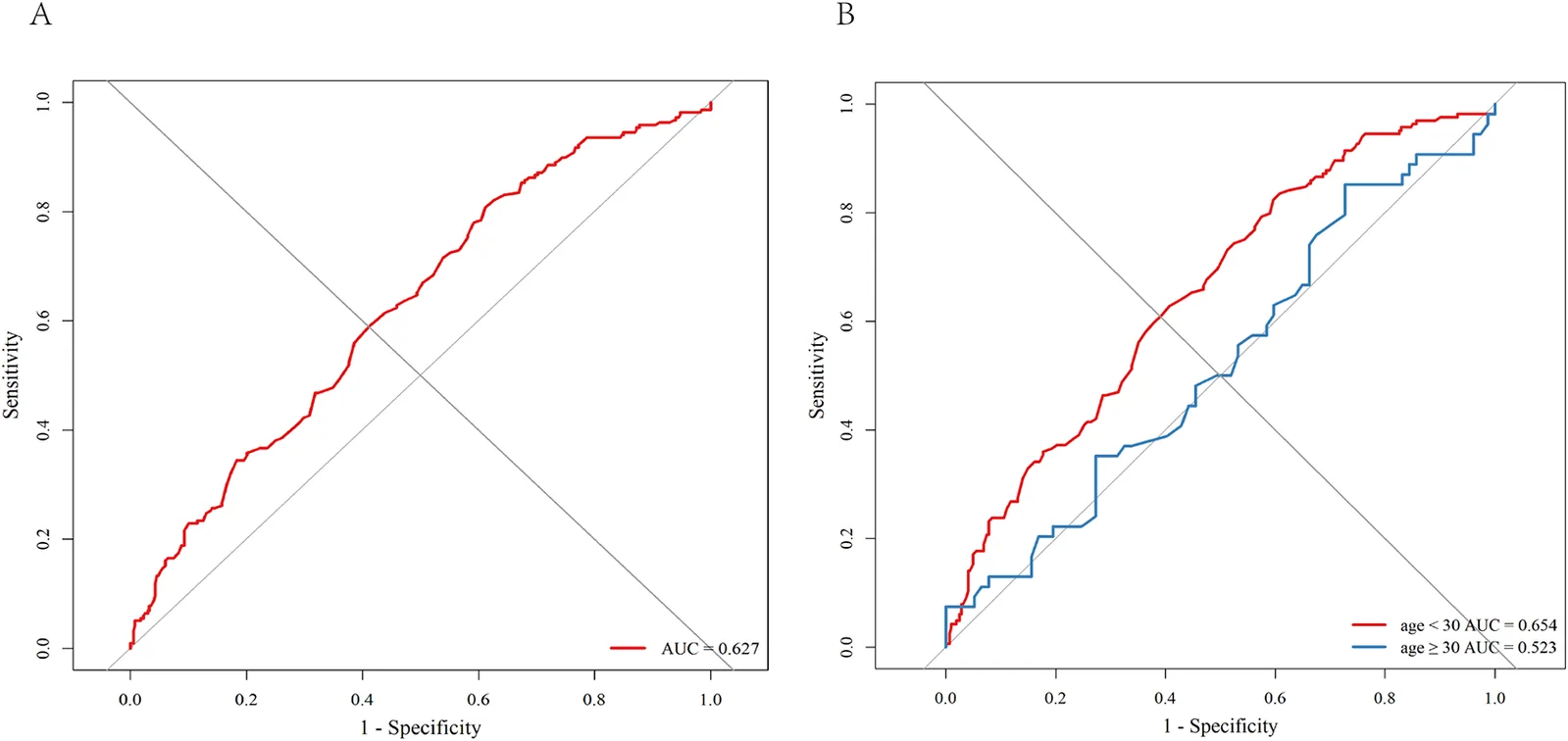
ROC analysis of lnRC for IR in women with PMOS. **(A)** Overall study population (AUC = 0.627). **(B)** Women aged <30 years (AUC = 0.654) and ≥30 years (AUC = 0.523). AUC, area under the curve.

**Table 4:** Age-stratified AUC comparison of lnRC for discriminating IR.

Group	n	AUC	ΔAUC	Best threshold	Sensitivity	Specificity	Delong-*p* value
Age < 30	486	0.654 (0.604-0.704)	0.131	2.434	0.823	0.404	0.024
Age ≥ 30	131	0.523 (0.422-0.624)		2.288	0.852	0.273	

### 3.5 Incremental discriminative information of lnRC beyond the base model

The incremental contribution of lnRC was examined by calculating IDI and NRI after its incorporation into the base model (**Table 5**). The expanded model yielded an NRI of 0.381 (95% CI: 0.165–0.590, *P* = 0.001) and an IDI of 0.025 (95% CI: 0.007–0.044; *P* = 0.005). These findings suggest that lnRC provided additional information for distinguishing women with and without IR within the study sample.

**Table 5:** Reclassification and discrimination improvement after adding lnRC to the baseline model for IR.

Model	NRI (95%)	P	IDI (95%)	P
Base				
Base+ lnRC	0.381 (0.165-0.597)	<0.001	0.025 (0.007-0.0439)	0.005

Base characteristics: age, BMI, SHBG, AMH, LH/FSH.

### 3.6 Sensitivity analyses

The stability of the main results was examined through several sensitivity analyses. The analyses were repeated after redefining IR as HOMA-IR ≥ 2.69. With this alternative criterion, lnRC continued to show a positive and stable relationship with IR (**Supplementary Table 1**). The age-specific estimates, multiplicative interaction, and ROC findings were also aligned with the primary results (**Supplementary Figure 1**, **Supplementary Tables 2**, **3**). Missing testosterone values (n = 126) were handled by multiple imputation. After imputation, the relationship between lnRC and IR remained broadly unchanged in the logistic regression models, and the age-stratified interaction pattern was also retained (**Supplementary Tables 4**, **5**). These additional analyses indicate that the main conclusions were not materially affected by testosterone imputation or by the alternative IR classification.

A sensitivity analysis was performed using 35 years as an alternative age cutoff (**Supplementary Table 6**). After covariate adjustment, each 1-unit increase in lnRC remained significantly associated with IR among women aged <35 years (OR = 1.773, 95% CI: 1.390–2.262; *P* < 0.001), whereas the corresponding estimate was smaller among women aged ≥35 years (OR = 1.167, 95% CI: 0.357–3.817; *P* = 0.799). The interaction between lnRC and age group was not statistically significant (*P* for interaction = 0.498). ROC analysis showed that the discriminative ability of lnRC for IR status was higher among women aged <35 years than among those aged ≥35 years [AUC: 0.630 (95% CI: 0.584–0.677) vs. 0.522 (95% CI: 0.287–0.757); DeLong *P* = 0.382] (**Supplementary Table 7**). After lnRC was added to the base model, the AUC increased from 0.854 to 0.868 in women aged <35 years (ΔAUC = 0.014; *P* = 0.046), whereas no increase in AUC was observed among women aged ≥35 years (**Supplementary Figure 2**, **Supplementary Table 8**).

No statistically significant interaction between BMI and lnRC was observed on either the multiplicative scale (interaction OR = 0.647, 95% CI: 0.231–1.815; *P* = 0.406) or the additive scale (RERI = 0.148, AP = 0.016, and SI = 1.019, all *P* > 0.05) (**Supplementary Table 9**). We additionally conducted an lnRC × age × BMI three-way interaction term (Estimates = 1.24, 95% CI: 0.58–2.65, *P* for interaction = 0.590), providing no evidence that BMI further modified the age-related heterogeneity in the lnRC–IR association (**Supplementary Table 10**).

## 4 Discussion

This study investigated the relationship between RC and IR among women with PMOS and found that lnRC remained positively associated with IR after adjustment for potential confounders. The RCS analysis also pointed to a nearly linear dose-response pattern, with the odds of IR rising as lnRC increased. Using the 30-year cutoff (34–37), the multiplicative interaction analysis suggested age-related heterogeneity, with a stronger association among women aged <30 years. The exploratory age-stratified ROC analysis yielded a significantly higher AUC in the younger subgroup. In addition, when lnRC was added to the metabolic and endocrine base model, both IDI and NRI improved significantly, indicating that lnRC may contribute information beyond conventional clinical indicators. Taken together, these findings highlight the potential clinical relevance of RC as an accessible adjunctive marker for metabolic assessment in women with PMOS, particularly those aged <30 years. Because RC can be readily calculated from routine lipid profiles, it may complement established metabolic and endocrine measures and help inform the need for further metabolic evaluation, particularly in younger women with PMOS. PMOS is now more often viewed as a heterogeneous metabolic-endocrine disorder, rather than only a reproductive condition (1, 39). Beyond ovulatory dysfunction and hyperandrogenism, women with PMOS often present with IR and several metabolic abnormalities, including dyslipidemia, impaired glucose metabolism, metabolic syndrome, and increased cardiometabolic risk (40, 41). Among these, IR may serve as an important bridge between metabolic and endocrine disturbances (42–44). Hyperandrogenism may further exacerbate insulin resistance and compensatory hyperinsulinemia, suppress hepatic SHBG synthesis, and dysregulate the hypothalamic–pituitary–ovarian (HPO) axis, thereby contributing to a self-reinforcing cycle of metabolic and reproductive dysfunction (14, 45–47). Dyslipidemia is also frequent in PMOS and affects approximately 50%–90% of women with this condition (48). Routine lipid parameters do not always reveal the underlying lipoprotein-related metabolic risk (49). Some women with apparently normal conventional lipid profiles may already have elevated apolipoprotein B (apoB), lipoprotein(a), or small dense LDL (50–53). In adolescents with obesity and PMOS, elevated fasting and postprandial apoB-containing remnant lipoproteins have also been reported, and these changes were closely related to impaired insulin metabolism and hyperandrogenaemia (40, 54). These findings indicate that remnant-related lipoprotein metabolism may be an overlooked part of metabolic dysfunction in PMOS, not simply another presentation of conventional dyslipidemia.

RC denotes the cholesterol content carried by triglyceride-rich remnant lipoproteins, including VLDL remnants, IDL, and chylomicron remnants (17, 18). Compared with TG, RC provides information on the cholesterol load of atherogenic remnant particles. In cardiovascular research, RC is often discussed as a lipid marker of residual cardiovascular risk. Higher RC has been related to a greater risk of atherosclerotic cardiovascular disease, myocardial infarction, and ischemic heart disease (55). Doi et al. further reported that including RC in conventional risk models improved reclassification for myocardial infarction and ischemic heart disease (56). However, the relevance of RC is not confined to cardiovascular outcomes and may also involve glucose metabolic disorders. Previous studies have linked higher RC levels with impaired glucose metabolism, new-onset hyperglycemia, and type 2 diabetes (25, 57–60). IR may be an important pathway associated with RC and metabolic disorders of glucose. In a Chinese population-based study, HOMA-IR significantly mediated the relationship between RC and diabetes mellitus, explaining 14.13% of the total association (25). Recent NHANES-based evidence also showed an independent positive relationship between HOMA-IR and RC. Even after stratification by LDL-C and TG levels, individuals with IR still had higher RC concentrations (61). In line with these observations, our study found that higher RC was linked to greater IR risk in women with PMOS. Because PMOS itself involves insulin-resistant metabolic disturbance, RC may reflect remnant lipoprotein-related dyslipidemia in this high-risk group.

The observed association between RC and IR is therefore biologically plausible. In PMOS, dysfunction of the liver and adipose tissue has been observed and may contribute to disturbances in lipid metabolism and dyslipidemia (14). During insulin-resistant states, hepatic VLDL production can increase, whereas clearance of VLDL and chylomicron remnants may become less efficient and both processes can favor the accumulation of cholesterol-enriched remnant particles (17). From another perspective, higher RC can be viewed as a circulating signal of lipid overload, residual cholesterol burden, and remnant lipoprotein accumulation (62). Such a lipid-metabolic background is often accompanied by lipotoxicity, inflammation, and impaired insulin signaling (63, 64). At the mechanistic level, excessive lipid exposure promotes bioactive lipid intermediates, including diacylglycerols and ceramides. Activation of the DAG/PKCϵ pathway may weaken insulin receptor signaling, while ceramides may disturb downstream Akt-mediated insulin signaling (65). Cholesterol overload can also trigger mitochondrial dysfunction, endoplasmic reticulum stress, oxidative stress, and inflammatory activation, all of which may disrupt glucose-insulin homeostasis (63, 64). Collectively, the available evidence suggests a potentially bidirectional interplay between RC and IR; however, our cross-sectional design precludes conclusions regarding causality or directionality.

Notably, our study suggests that age may modify the association between RC and IR. This result adds to the current understanding of age-related metabolic changes in PMOS, as it is a dynamic disorder whose endocrine and metabolic manifestations may change across the reproductive lifespan (66, 67). Some studies have reported that metabolic burden becomes more pronounced with advancing age (68, 69). Other evidence shows that glucose homeostasis, fasting insulin levels, and insulin-resistance indices, including HOMA-IR, may not necessarily worsen even when BMI and WC increase (35). Nevertheless, IR and related cardiometabolic abnormalities can still persist throughout the course of PMOS (5, 70, 71), and affected women may develop type 2 diabetes at a younger age than women without PMOS (72). These observations suggest that age-specific metabolic changes in PMOS are heterogeneous rather than uniformly progressive. Our findings further indicate that age may modify the lnRC–IR relationship. The significant multiplicative interaction indicated that the association was stronger among women aged <30 years and attenuated among those aged ≥30 years. Consistent with this finding, lnRC exhibited better discriminative performance for IR in the younger subgroup. Several previous studies of PMOS have used an age stratification of <30 and ≥30 years to evaluate age-related differences in clinical phenotypes, insulin resistance, lipid metabolism, and cardiometabolic characteristics (34–37). These studies suggest that the endocrine and metabolic phenotypes of PMOS vary with age and provide a pragmatic rationale for using 30 years as a clinically interpretable cutoff in stratified analyses. A sensitivity analysis using 35 years as an alternative cutoff showed a similar pattern in the subgroup-specific ORs, with a stronger association in the younger group. To assess whether BMI modified the lnRC–IR association, we examined the BMI × lnRC interaction on both the multiplicative and additive scales and found no statistically significant interaction on either scale. We then tested the lnRC × age × BMI three-way interaction and found no evidence that the observed age-related variation was further modified by BMI levels. Taken together, these complementary analyses provide suggestive evidence that age may modify the lnRC–IR relationship, which appears more pronounced in younger women with PMOS and is not clearly explained by BMI. This pattern highlights age as a potentially important source of heterogeneity and supports further age-stratified evaluation of RC in larger prospective cohorts with more balanced age and BMI distributions.

Considering the close relationship among PMOS, IR, and dyslipidemia, especially the early appearance of atherogenic dyslipidemia in younger PMOS patients, RC may help capture insulin-resistant dyslipidemia in this subgroup. This interpretation is supported by previous studies showing increased levels of triglyceride-rich lipoprotein remnants and apoB-containing remnant particles in adolescents and younger women with PMOS. Since RC reflects the cholesterol load carried by remnant lipoproteins, elevated RC levels among younger women with PMOS may indicate the accumulation of remnant lipoproteins and lipid metabolism disorders associated with IR. Several studies involving lipid-lowering drugs or dietary interventions that evaluated changes in remnant-related lipid measures together with indices of IR and glucose metabolism. Regarding fibrates, which are primarily used to lower TC and triglyceride-rich remnant lipoproteins, Nakamura et al. conducted a randomized crossover trial involving 60 patients with hypertriglyceridemia and stable coronary artery disease. Both pemafibrate and bezafibrate significantly reduced RemL-C levels and improved markers of glucose metabolism and IR. A dietary intervention study also reported supportive findings. Van Hees et al. conducted a 10-week energy-restricted dietary intervention in individuals with obesity and observed a significant reduction in fasting RLP-C. The reduction in RLP-C was associated with weight loss, amelioration in HOMA-IR, and the fat content of the prescribed diet. Overall, these studies suggest that lipid-lowering and dietary interventions may reduce RemL-C/RLP-C and alleviate IR and glucose metabolism. In women with PMOS, Vine et al. conducted a 12-week randomized, open-label exploratory trial involving 29 women aged 18–30 years with PMOS and metabolic syndrome. Participants were randomized to receive fish oil, metformin, or a combination of fish oil and metformin. In the combination group, fasting triglycerides decreased by more than 40%, fasting ApoB48 decreased by approximately 40%, and HOMA-IR decreased numerically by approximately 32%. However, the between-group differences in fasting insulin, fasting glucose, 2-h glucose during the OGTT, and HOMA-IR were not statistically significant. Nevertheless, given its small sample size and the established insulin-sensitizing effect of metformin, it remains uncertain whether lowering remnant-related lipoproteins independently ameliorates IR. At the same time, we identified randomized trial evidence that does not fully support this hypothesis. The ANCHOR study was a 12-week, multicenter, randomized, double-blind, placebo-controlled phase III trial showed that Icosapent ethyl significantly reduced RLP-C, triglycerides, VLDL-C, ApoB, and other triglyceride-rich lipoprotein measures. However, no statistically significant placebo-adjusted changes were observed in HOMA-IR. The intervention studies described above primarily assessed RLP-C, RemL-C, or ApoB48- or ApoB100-containing lipoproteins, they capture overlapping aspects of triglyceride-rich lipoprotein and remnant metabolism. Therefore, these studies provide indirect biological plausibility for a dynamic relationship between RC and IR. However, the evidence remains limited and inconsistent. No adequately powered randomized controlled trial has examined whether lowering RC improves IR in women with PMOS, leaving this question unresolved.

To our knowledge, this is the first observational study that provides evidence of an association between RC and IR in women with PMOS and suggests that age may modify this relationship, which appeared stronger in younger women. Given the practical limitations of fasting insulin measurement and derived IR indices in routine clinical care, RC may provide readily accessible, complementary information for metabolic assessment, as it can be calculated from a standard lipid profile. However, its modest standalone discriminatory performance means that it should be interpreted alongside established metabolic and endocrine measures. In this context, RC may serve as an additional indicator of metabolic disturbance and support consideration of further metabolic evaluation. These findings highlight younger women with PMOS as an important subgroup for further investigation and may help generate testable hypotheses and inform the design and target populations of future longitudinal and interventional studies.

Nevertheless, these findings should be interpreted in light of several constraints. First, its cross-sectional design and single-time-point measurements of RC, glucose, insulin, and related lipid parameters preclude assessment of temporal ordering, longitudinal changes, and causal direction between RC and IR. Accordingly, this study cannot determine whether lowering RC improves IR. Second, although the exclusion criteria reduced the potential influence of recent treatments, incomplete information on metformin, combined oral contraceptives, lipid-lowering therapy, weight-management interventions, diet, and physical activity leaves open the possibility of residual confounding. Third, the single-center sample comprised only reproductive-aged women with PMOS and had limited geographic, ethnic, age, and body-weight diversity, which may restrict the generalizability of our findings to broader populations. Moreover, the sensitivity analysis using the 35-year age cutoff and the BMI interaction analyses were limited by small and imbalanced subgroups, resulting in imprecise estimates and limited power to detect effect modification. Future multicenter prospective studies should incorporate repeated measurements, broader population representation, and standardized collection of treatment and lifestyle data. Appropriately designed intervention trials are also needed to determine whether lowering RC improves IR.

## 5 Conclusion

Among women with PMOS, higher RC levels were positively associated with IR, with a stronger relationship among women aged <30 years. The multiplicative interaction further supported age-related effect modification. LnRC also showed greater discriminatory ability in the younger subgroup. Readily calculable from routine lipid profiles, RC may serve as a practical adjunct to established metabolic and endocrine measures, particularly in the metabolic assessment of younger women with PMOS.

## Data Availability

The raw data supporting the conclusions of this article will be made available by the authors, without undue reservation.
