# Supplementary material for: Age-specific association between remnant cholesterol and insulin resistance in women with polyendocrine metabolic ovarian syndrome
Source: Front Endocrinol (Lausanne). 2026 Sep 30;17:1932335. doi: 10.3389/fendo.2026.1932335 (PMC13661191; doi:10.3389/fendo.2026.1932335)
Supplement: Supplementary file 1 [file DataSheet1.docx]

Supplementary Table 1. Sensitivity analysis of the association between lnRC and IR (defined by HOMA-IR ≥ 2.69).

|  | Crude model | | Model 1 | | Model 2 | | Model 3 | |
| --- | --- | --- | --- | --- | --- | --- | --- | --- |
| Categories | OR  (95% CI) | *P* value | OR  (95% CI) | *P* value | OR  (95% CI) | *P* value | OR  (95%CI) | *P* value |
| lnRC | 1.74 (1.37-2.22) | < 0.001 | 1.72 (1.35-2.19) | < 0.001 | 1.66 (1.27-2.17) | < 0.001 | 1.58 (1.17-2.12) | 0.003 |
| lnRC |  |  |  |  |  |  |  |  |
| (quartiles) |  |  |  |  |  |  |  |  |
| Q1 (≤ 2.23) | 1.00 (reference) | | 1.00 (reference) | | 1.00 (reference) | | 1.00 (reference) | |
| Q2 (2.23-2.81) | 1.83 (1.08-3.1) | 0.02 | 1.82 (1.07-3.08) | 0.026 | 2.41 (1.28-4.53) | 0.006 | 2.24 (1.12-4.47) | 0.023 |
| Q3 (2.81-3.18) | 2.45 (1.46-4.1) | < 0.001 | 2.41 (1.44-4.05) | < 0.001 | 2.46 (1.32-4.57) | 0.004 | 2.66 (1.31-5.39) | 0.007 |
| Q4 (≥ 3.18) | 3.47 (2.09-5.78) | < 0.001 | 3.40 (2.04-5.66) | < 0.001 | 3.03 (1.65-5.56) | < 0.001 | 2.92 (1.46-5.84) | 0.002 |
| *P* - trend | < 0.001 | | < 0.001 | | < 0.001 | | 0.003 | |

Crude model: unadjusted

Model 1: adjusted for age
Model 2: adjusted for age, BMI
Model 3: adjusted for age, BMI, Testosterone

Supplementary Table 2. Age-stratified sensitivity analysis of the association between lnRC and IR (defined by HOMA-IR ≥ 2.69).

| Age subgroup | Total | Events (%) | OR (95% CI) | *P* value | *P* for interaction |
| --- | --- | --- | --- | --- | --- |
| Age < 30 | 486 | 154 (31.7) | 2.089 (1.552-2.812) | < 0.001 | 0.040 |
| Age ≥ 30 | 131 | 50 (38.2) | 1.156 (0.716-1.866) | 0.554 |  |

Supplementary Figure 1. ROC analysis of lnRC for IR (defined by HOMA-IR ≥ 2.69) in women with PMOS. (A) Overall study population (AUC = 0.631). (B) Women aged <30 years (AUC = 0.659) and ≥30 years (AUC = 0.533). AUC, area under the curve; lnRC, natural logarithm of remnant cholesterol.

Supplementary Table 3. Age-stratified AUC comparison of lnRC for discriminating IR (defined by HOMA-IR ≥ 2.69).

| Group | n | AUC | ∆AUC | Best cutoff | Sensitivity | Specificity | Delong-*P* value |
| --- | --- | --- | --- | --- | --- | --- | --- |
| Age < 30 | 413 | 0.659 (0.608-0.709) | 0.126 | 2.388 | 0.392 | 0.857 | 0.035 |
| Age ≥ 30 | 204 | 0.533 (0.429-0.638) |  | 3.201 | 0.728 | 0.38 |  |

Supplementary Table 4. Logistic regression analysis after multiple imputation of testosterone.

|  | Model 3 | |
| --- | --- | --- |
| Categories | OR (95% CI) | *P* value |
| lnRC | 1.69 (1.30-2.19) | < 0.001 |
| lnRC |  |  |
| (quartiles) |  |  |
| Q1 (≤ 2.23) | 1.00 (reference) | |
| Q2 (2.23-2.81) | 2.43 (1.33-4.45) | 0.004 |
| Q3 (2.81-3.18) | 2.72 (1.49-4.98) | 0.001 |
| Q4 (≥ 3.18) | 3.38 (1.86-6.11) | < 0.001 |
| *P* - trend | < 0.001 | |

Model 3: adjusted for age, BMI, Testosterone

Supplementary Table 5. Age-stratified analysis after multiple imputation of testosterone.

| Group | Total | OR (95% CI) | *P* value | *P* for interaction |
| --- | --- | --- | --- | --- |
| Age < 30 | 486 | 2.432 (1.783-3.318) | < 0.001 | 0.048 |
| Age ≥ 30 | 131 | 1.198 (0.638-2.248) | 0.574 |  |

Based on logistic regression Model 3 (adjusted for age, BMI, Testosterone)

Supplementary Table 6. Sensitivity analysis of the association between lnRC and IR by age group (<35 vs. ≥35 years).

| Age subgroup | Total | Events (%) | OR (95% CI) | *P* value | *P* for interaction |
| --- | --- | --- | --- | --- | --- |
| Age < 35 | 456 | 163 (35.7) | 1.773 (1.390-2.262) | < 0.001 | 0.498 |
| Age ≥ 35 | 21 | 12 (57.1) | 1.167 (0.357-3.817) | 0.799 |  |

Based on logistic regression Model 3.

Supplementary Table 7. Sensitivity analysis of the age-stratified discriminative performance of lnRC for IR (<35 vs. ≥35 years).

| Group | n | AUC (95%CI) | ∆AUC | Best threshold | sensitivity | specificity | Delong-*P* value |
| --- | --- | --- | --- | --- | --- | --- | --- |
| Age < 35 | 586 | 0.630 (0.584-0.677) | 0.108 | 2.857 | 0.583 | 0.613 | 0.382 |
| Age ≥ 35 | 31 | 0.522 (0.287-0.757) |  | 3.201 | 0.417 | 0.789 |  |

Supplementary Figure 2. Sensitivity analysis: age-stratified ROC analysis of lnRC for IR using a 35-year cutoff.

Supplementary Table 8. Sensitivity analysis: incremental discriminative value of lnRC beyond the base model by age group (<35 vs. ≥35 years).

| Group | | AUC (95%CI) | ∆AUC | Best threshold | sensitivity | specificity | Delong-*P* value |
| --- | --- | --- | --- | --- | --- | --- | --- |
| Age < 35 | Base model | 0.854 (0.811-0.897) |  | 0.467 | 0.699 | 0.876 |  |
|  | +lnRC | 0.868 (0.828-0.908) | 0.014 | 0.322 | 0.832 | 0.758 | 0.0464 |
| Age ≥ 35 | Base model | 0.889 (0.655-1.000) |  | 0.497 | 1.000 | 0.750 |  |
|  | +lnRC | 0.889 (0.655-1.000) | 0 | 0.488 | 1.000 | 0.750 | 1 |

Supplementary Table 9. Multiplicative and additive interactions between BMI and lnRC in relation to IR.

| Measures | Estimates (95% CI) | P-value |
| --- | --- | --- |
| Multiplicative interaction |  |  |
| Multiplicative scale | 0.647 (0.231-1.815) | 0.406 |
| Additive interaction |  |  |
| RERI (95% CI) | 0.148 (-8.408-7.281) | 0.960 |
| AP (95% CI) | 0.016 (-0.969-0.512) | 0.960 |
| SI (95% CI) | 1.019 (0.465-2.249) | 0.976 |

Supplementary Table 10. Three-way multiplicative interaction among lnRC, age, and BMI in relation to IR.

|  | Estimates (95% CI) | P for Interaction |
| --- | --- | --- |
| 3-Way Interaction  (lnRC × age× BMI) | 1.24 (0.58-2.65) | 0.59 |
